# Deep graph learning of multimodal brain networks defines treatment-predictive signatures in major depression

**DOI:** 10.1038/s41380-025-02974-6

**Published:** 2025-03-31

**Authors:** Yong Jiao, Kanhao Zhao, Xinxu Wei, Nancy B. Carlisle, Corey J. Keller, Desmond J. Oathes, Gregory A. Fonzo, Yu Zhang

**Affiliations:** 1 Department of Bioengineering, Lehigh University, Bethlehem, PA USA; 2 Department of Electrical and Computer Engineering, Lehigh University, Bethlehem, PA USA; 3 Department of Psychology, Lehigh University, Bethlehem, PA USA; 4 Department of Psychiatry and Behavioral Sciences, Stanford University School of Medicine, Stanford, CA USA; 5 Wu Tsai Neurosciences Institute, Stanford University, Stanford, CA USA; 6 Veterans Affairs Palo Alto Healthcare System, and the Sierra Pacific Mental Illness, Research, Education, and Clinical Center (MIRECC), Palo Alto, CA USA; 7 Center for Brain Imaging and Stimulation, Department of Psychiatry, University of Pennsylvania Perelman School of Medicine, Philadelphia, PA USA; 8 Center for Neuromodulation in Depression and Stress, Department of Psychiatry, University of Pennsylvania Perelman School of Medicine, Philadelphia, PA USA; 9 Penn Brain Science, Translation, Innovation, and Modulation Center, University of Pennsylvania Perelman School of Medicine, Philadelphia, PA USA; 10 Departments of Neurology, Neurosurgery, Bioengineering and Neuroscience, University of Pennsylvania Perelman School of Medicine, Philadelphia, PA USA; 11 Center for Psychedelic Research and Therapy, Department of Psychiatry and Behavioral Sciences, Dell Medical School, The University of Texas at Austin, Austin, TX USA

**Keywords:** Predictive markers, Depression, Biological techniques, Neuroscience

## Abstract

Major depressive disorder (MDD) presents a substantial health burden with low treatment response rates. Predicting antidepressant efficacy is challenging due to MDD’s complex and varied neuropathology. Identifying biomarkers for antidepressant treatment requires thorough analysis of clinical trial data. Multimodal neuroimaging, combined with advanced data-driven methods, can enhance our understanding of the neurobiological processes influencing treatment outcomes. To address this, we analyzed resting-state fMRI and EEG connectivity data from 130 patients treated with sertraline and 135 patients with placebo from the Establishing Moderators and Biosignatures of Antidepressant Response in Clinical Care (EMBARC) study. A deep learning framework was developed using graph neural networks to integrate data-augmented connectivity and cross-modality correlation, aiming to predict individual symptom changes by revealing multimodal brain network signatures. The results showed that our model demonstrated promising prediction accuracy, with an R^2^ value of 0.24 for sertraline and 0.20 for placebo. It also exhibited potential in transferring predictions using only EEG. Key brain regions identified for predicting sertraline response included the inferior temporal gyrus (fMRI) and posterior cingulate cortex (EEG), while for placebo response, the precuneus (fMRI) and supplementary motor area (EEG) were critical. Additionally, both modalities identified the superior temporal gyrus and posterior cingulate cortex as significant for sertraline response, while the anterior cingulate cortex and postcentral gyrus were common predictors in the placebo arm. Additionally, variations in the frontoparietal control, ventral attention, dorsal attention, and limbic networks were notably associated with MDD treatment. By integrating fMRI and EEG, our study established novel multimodal brain network signatures to predict individual responses to sertraline and placebo in MDD, providing interpretable neural circuit patterns that may guide future targeted interventions. Trial Registration: Establishing Moderators and Biosignatures of Antidepressant Response for Clinical Care for Depression (EMBARC) ClinicalTrials.gov Identifier: NCT#01407094.

## Introduction

Major depressive disorder (MDD) remains a major global mental health concern, affecting millions of people. Despite the widespread use of serotonin reuptake inhibitors and other antidepressants, their effectiveness varies substantially, leaving many patients without sufficient relief [1]. This variability underscores the urgent need to understand the biological and psychological mechanisms driving different treatment responses. Personalized treatment strategies, considering the heterogeneity of MDD, are becoming increasingly important [2, 3]. Emerging evidence suggests that brain network (or connectivity) variations may capture pivotal information associated with treatment effects [4, 5]. Understanding these complex variations is vital for developing more effective, personalized therapeutic strategies for MDD.

In neuropsychiatric research, functional magnetic resonance imaging (fMRI) and electroencephalography (EEG) are essential for studying the neurobiology of MDD [6]. By measuring hemodynamic responses to neural activity, fMRI provides spatially high-resolution images of brain function, identifying regions implicated in psychiatric disorders [7]. In contrast, EEG records electrical oscillations from neural activity, offering superior temporal resolution for capturing network dynamics [8]. Together, these modalities enhance our understanding of the complex neural circuits associated with MDD, supporting the advancement of more tailored therapeutic interventions.

The fusion of multimodal brain imaging techniques is gaining popularity in mental disorder analysis, since it allows for a multidimensional analysis, providing comprehensive insights into the interrelationships between various imaging modalities. This integrative technique offers a more reliable foundation for precision diagnosis and prognosis, enhancing the analytical capabilities of each technique. Recent studies have successfully used multimodal fusion to predict and understand various mental health conditions. For instance, utilizing complementary information from MRI, PET, assessment metrics, and demographic data has shown promise for improved diagnoses of Alzheimer’s disease and autism [9]. Another recent study employed multimodal fusion of functional and structural data to provide a comprehensive view of longitudinal changes in brain patterns [10]. Integrating functional and structural connectivity from fMRI and diffusion tensor imaging emphasized dynamic inter-modal dependencies [11]. Furthermore, coupled tensor/matrix factorization was used to extract joint latent components from MEG and fMRI data, revealing fine-grained spatiotemporal brain dynamics and improving understanding of brain function and development in adolescents [12]. A recent study on MDD identified common and unique structural and functional connectivity coupling changes, highlighting the structural and functional connectivity coupling changes, highlighting the vulnerability of these connections to external stressors and adverse behaviors [13]. Despite these advances, there is a lack of research employing the fusion of fMRI and EEG to predict antidepressant outcomes. Combining EEG’s temporal resolution with fMRI’s spatial detail at the brain network level may offer a comprehensive view of neural circuits, enhancing our understanding of the neurobiological basis of MDD and its interaction with antidepressant treatment.

Recent advancements in machine learning and artificial intelligence have demonstrated potential in identifying brain biomarkers for more precise mental health interventions [14, 15]. The human brain can be modeled as a complex network comprising numerous spatially distributed but functionally interconnected brain regions [16]. Graph neural networks (GNNs) [17], a type of deep learning models, are particularly suited for analyzing brain functional architecture due to their ability to handle complex node interactions. This unique advantage has led to their increasing application in studying mental disorders through brain network modeling [11, 18–22]. Nevertheless, very few studies employing GNNs have focused on multi-modality brain network analysis for predicting treatment responses leveraging clinical trial data.

Utilizing data from the Establishing Moderators and Biosignatures of Antidepressant Response in Clinical Care (EMBARC) study, a large neuroimaging-coupled randomized clinical trial, we propose a novel deep learning framework integrating fMRI and EEG connectivity to establish robust biomarkers for predicting antidepressant and placebo treatment responses in MDD. This framework augments the functional connectivity of both modalities and constructs brain graphs for pre-treatment fMRI and EEG data, respectively. It subsequently employs GNNs to capture the spatial dependencies within each modality, thereby optimizing spatial characteristics. By maximizing the correlation between latent features, our approach harnesses complementary information through modality fusion to predict treatment outcomes. An interpretability analysis conducted based on the trained model parameters further identifies critical biomarkers from both brain region and connectivity perspectives, advancing our comprehension of neural circuits that dictate response to antidepressant treatment.

## Methods and materials

### Clinical trial dataset

Our study utilized data from the EMBARC study [1], a comprehensive, neuroimaging-integrated, placebo-controlled, randomized clinical trial focusing on depression. Participants aged 18 to 65 were enrolled from four study locations: Massachusetts General Hospital, University of Texas Southwestern Medical Center, University of Michigan, and Columbia University. In this double-blind trial, individuals diagnosed with MDD were randomly assigned to receive either an eight-week treatment of sertraline or placebo. Out of 309 patients diagnosed with MDD, 296 met the inclusion criteria and received treatment. For subjects who have both fMRI and EEG data, there are 130 from the sertraline arm (38 male, 92 female) and 135 from the placebo arm (50 male, 85 female) after preprocessing. The primary outcome was measured using the 17-item Hamilton Rating Scale for Depression (HAMD_17_) [23] at multiple time points (baseline, weeks 1, 2, 3, 4, 6, and 8). Missing HAMD_17_ scores at week 8 were estimated through Bayesian regression methods [24] using baseline HAMD_17_, week 1 HAMD_17_, week 2 HAMD_17_, week 3 HAMD_17_, week 4 HAMD_17_, week 6 HAMD_17_, baseline Quick Inventory of Depressive Symptoms (QIDS) total score, baseline Mood and Symptom Questionnaire subscale scores for Anxious Arousal, Anhedonic Depression, and General Distress, Snaith-Hamilton Pleasure Scale (SHAPS) total score, age, years of education, gender, and Wechsler Abbreviated Scale of Intelligence (WASI) t-scores for Vocabulary and Matrix Reasoning. Treatment outcome was measured by the change in HAMD_17_ scores from the baseline to week 8.

### fMRI acquisition and preprocessing

Resting-state functional magnetic resonance imaging (rs-fMRI) scans were conducted using T2* weighted images via a single-shot gradient echo-planar pulse sequence. Each session lasted eight minutes using parameter settings: repetition time 2000 ms, echo time 28 ms, matrix size 64 × 64, voxel size 3.2 × 3.2 × 3.1 mm^3^. More details of the fMRI acquisition can be found in [25].

The rs-fMRI data were preprocessed using the fMRIPrep pipeline [26]. This involved correcting the T1 weighted images for intensity nonuniformity and skull stripping, followed by spatial normalization via nonlinear registration to a T1w reference [27]. Brain tissues, including cerebrospinal fluid, white matter, and grey matter, were segmented using FSL from the brain-extracted T1 weighted images [28]. Fieldmap information was utilized to correct distortions in the echo-planar imaging, improving the co-registration with the anatomical reference. The BOLD signals were aligned to the T1-weighted images using boundary-based registration with nine degrees of freedom to address remaining distortions [29]. Head-motion parameters were estimated using MCFLIRT (FSL), and the BOLD signals were subjected to slice-time correction, susceptibility distortion correction, and were resampled into MNI152NLin2009cAsym space. Motion artifacts were removed using ICA-AROMA [30], and following spatial smoothing with a 6 mm FWHM Gaussian kernel, the data underwent quality control for head motion.

### EEG acquisition and preprocessing

Resting-state EEG (rs-EEG) data were recorded from each of the four study sites (Columbia University: 72-channel BioSemi system at 256 Hz sampling rate, McLean Hospital: 129-channel Geodesic Net system at 250 Hz, University of Michigan: 60-channel NeuroScan Synamp system at 250 Hz, University of Texas Southwestern Medical Center: 62-channel NeuroScan Synamp system at 250 Hz). Amplifier calibrations were consistently performed across all sites. The rs-EEG recording protocol consisted of four two-minute segments—two with eyes closed and two with eyes open, executed in a counterbalanced sequence. Participants were advised to minimize blinking and eye movements, maintaining fixation on a central cross during the eyes-open condition.

The rs-EEG data was processed offline using a fully automated artifact rejection pipeline in EEGLAB [2]. Initially, EEG signals were downsampled to a frequency of 250 Hz, followed by the elimination of the 60 Hz AC line noise [31]. Subsequently, a high-pass filter with a cutoff frequency of 0.01 Hz was employed to filter out low-frequency elements that were not physiological. The next stages involved the identification and removal of epochs and channels that did not meet specific threshold criteria for magnitude and spatial correlation, respectively. Participants with an excess of 20% problematic channels were excluded. For channels with compromised signals, a reconstruction was carried out using spherical spline interpolation based on signals from adjacent channels [32]. To further cleanse the data, independent component analysis was applied to remove additional artifacts like those caused by scalp muscles, eye movements, and ECG [33]. After artifact rejection, 54 EEG channels common to all four study sites were identified and extracted for each subject. The EEG signals were then standardized to a common average reference. Lastly, the signals were divided into four key frequency bands: theta (4–7 Hz), alpha (8–12 Hz), beta (13–30 Hz), and low gamma (31–50 Hz) for further analysis.

Source localization using the Brainstorm toolbox [34] with a minimum-norm estimation [35] was then conducted to convert channel-space EEG into source-space signals over 3003 vertices. This conversion was achieved through a three-layer boundary element head model, comprising scalp, skull, and cortical surface, calculated using the OpenMEEG plugin [36], which utilizes the FreeSurfer average brain template [37]. The model facilitated the generation of 3003 dipoles with free orientations. A lead-field matrix, critical for linking the activity of these dipoles to EEG data, was subsequently derived from the boundary element model. Principal component analysis was then applied to condense the three-dimensional estimated source signal at each vertex into a one-dimensional time series of the principal component.

### Functional connectivity calculation

For rs-fMRI, regional time series was first computed by averaging the preprocessed voxel-level BOLD signals into time series for 100 regions of interest (ROIs) defined by the Schaefer parcellation [38]. Functional connectivity was then calculated as Pearson correlation coefficient between the time series of every pair of ROIs. The resulting connectivity values were *z*-score normalized and then subjected to Fisher’s *r*-to-*z* transformation to improve normality.

For rs-EEG, power envelope connectivity (PEC) was calculated in preference to other metrics such as coherence or phase lag index, as PEC not only has shown promise in mitigating spurious correlations caused by volume conduction, a common issue in EEG that can produce misleading connectivity patterns between adjacent channels [39, 40], but also demonstrates superior stability in resting-state analyses as power envelopes exhibit more consistent temporal characteristics than instantaneous phase relationships [41]. More importantly, PEC’s focus on power envelope fluctuations provides a more compatible temporal framework for integration with fMRI’s hemodynamic responses, as it captures slow temporal dynamics of neural oscillation amplitudes (typically <1 Hz) [42]. This temporal correspondence has been empirically validated by multiple studies showing significant spatial overlap between PEC-derived networks and fMRI connectivity patterns [43]. PEC addresses the volume conduction problem by focusing on the oscillatory power of signals, rather than their raw phase or amplitude, which are more susceptible to such artifacts. Specifically, Hilbert transform was first used to convert source estimates into analytical time series, followed by orthogonalizing the analytical time series of each pair of brain signals to eliminate zero-phase-lag correlation. Unlike phase lag index which may discard genuine zero-phase-lag connectivity, PEC’s orthogonalization approach maintains sensitivity to physiologically meaningful synchronization while effectively eliminating artifactual correlations [44]. The power envelopes were then obtained by squaring these orthogonalized analytical signals. A logarithmic transformation was subsequently applied to refine the data and enhance the normality of the measurements. PEC was then calculated using Pearson’s correlation coefficient between the log-transformed power envelopes of each brain region pair. Similar to fMRI, EEG connectivity features were extracted based on the Schaefer parcellation. The resulting connectivity values were *z*-score normalized before undergoing Fisher’s *r*-to-*z* transformation to improve normality and ensure comparability across modalities.

### Data augmentation

Data augmentation is widely adopted in deep learning as a strategy to enhance performance and prevent overfitting. To increase the sample size for more robust multimodal fusion and prevent overfitting, we employed Common Orthogonal Basis Extraction (COBE) [45], a well-established algorithm in group component analysis, to augment functional connectivity data for both fMRI and EEG. COBE is adept at extracting a common basis from multi-block datasets. By removing the common components from the original functional connectivity of fMRI or EEG, individualized features are isolated. These individualized features, as newly generated data, retain essential discriminatory information related to their original functional connectivity, thereby forming the augmented data in this study. Additional details about the augmentation process are provided in Supplementary S1.

### Deep learning of multimodal brain network signatures

**Fig. 1:**
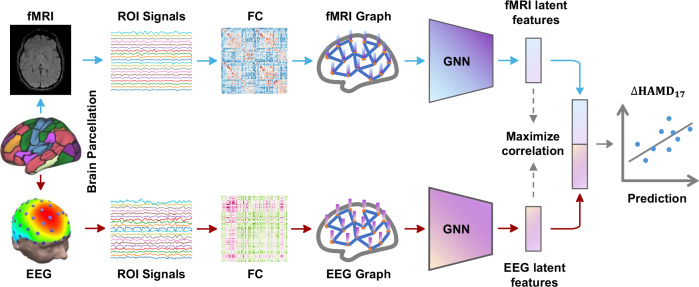
The flowchart of the framework. Functional connectivity (FC) features derived from fMRI and EEG are firstly obtained based on parcellation and subsequently encoded via two parallel GNNs. The correlation between these multimodal representations is then maximized to enhance their compatibility for modality fusion. Subsequently, these highly correlated latent variables are concatenated and fed into a multilayer perceptron (MLP) to predict changes in HAMD_17_ score. More detailed implementation aspects of the architecture are available in Supplementary Figure S2.

We developed a GNN-based deep learning model to integrate multimodal brain networks from fMRI and EEG, identifying robust and powerful brain signatures indicative of antidepressant response. Our model consists of two main components: 1) utilizing parallel GNNs to encode the connectivity features of fMRI and EEG, performing spatial optimization; 2) training pairs of fMRI and EEG weights to ensure that the linear combination of node features maximizes the correlation in resulting latent features between these two modalities to achieve effective modality fusion. The overview of the framework is illustrated in Fig. 1. GNNs are particularly effective for processing graph-structured data due to their capacity to encapsulate the intricate relationships among nodes [46, 47]. Methods and implementation details are provided in Supplementary S2 and S3.

### Brain region and connectivity importance evaluation

Besides the novel multimodal graph fusion strategy, our model also offers interpretable brain network patterns, advancing the understanding of neurobiological mechanisms underlying treatment effects. We used two learnable weight matrices (see Supplementary S2) in our model function as mappings for maximizing the correlation between fMRI and EEG latent features. These matrices quantify the contributions of different ROIs to the fMRI-EEG association as self-learned model parameters during end-to-end training, thus providing an intuitive reflection of the brain regional importance in modality fusion. We integrated these pairs of vectors into a singular pair using the weights in the aggregation block, followed by absolute value computation and normalization. An average was then computed over ten folds in the optimal run as the evaluation of the brain region importance.

In addition, the adjacency matrix of the graphs was constructed by calculating similarities between node pairs in real-time, reflecting the inherent associations among ROIs. However, it may not represent the optimal topology for subsequent modality fusion and final predictions. To address this, we introduced scaling matrices for fMRI and EEG, enabling dynamic estimation and adjustment of connection strength between ROI pairs during end-to-end training for each modality. This approach captures and refines crucial connectivity patterns tailored for modality fusion and final prediction, rather than solely relying on similarity measures. We assessed the significance of connections by averaging the matrix values from the most predictive runs for each modality in both treatment arms. It is important to note that the learnable weight matrices and the scaling matrices serve distinct but complementary roles in our model. The learnable weight matrices are used for selecting salient brain regions for modality fusion, while the scaling matrices focus on interpreting optimized dependencies within graph-structured unimodal data.

## Results

### Multimodal prediction of treatment outcome

**Fig. 2:**
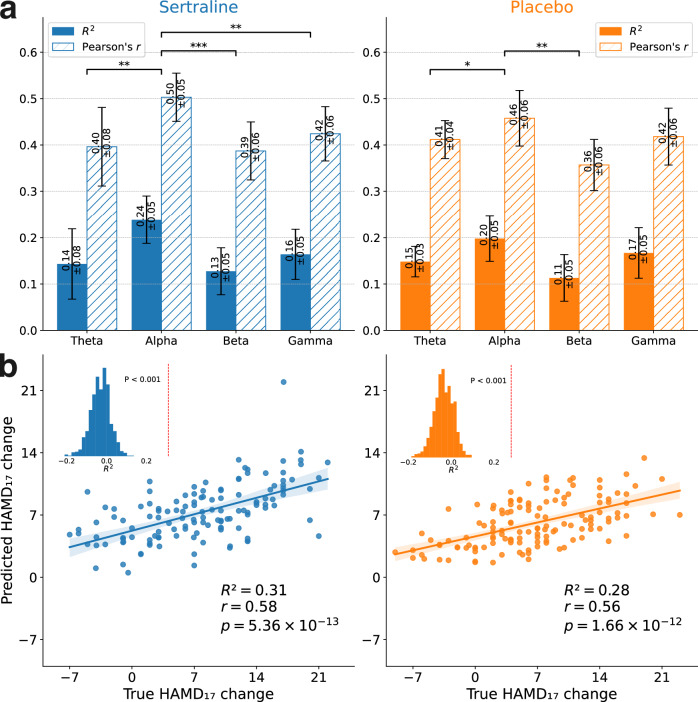
Performance of treatment outcome prediction for the sertraline (blue) and placebo (orange) arms, respectively. **a** Prediction results across four EEG bands under our deep learning-based multimodal analytical framework. **b** The optimal run of predictive outcomes using alpha band EEG in conjunction with fMRI. The statistical significance of predictive accuracy was validated through 1000 random permutation tests with Ppermutation<0.001 for both sertraline and placebo. Each point represents a patient in the test set.

We hypothesize that different EEG frequency bands contain distinct discriminative information relevant to treatment prediction. Therefore, instead of using a single unified model, we combined EEG data from each frequency band with fMRI to train separate models. Figure 2a shows the predictive results of two treatment arms across four EEG bands combined with fMRI, derived from ten independent runs of 10-fold cross-validation using various seeds for model parameter initialization. Combining fMRI with EEG in the theta, alpha, and beta bands show notable predictiveness. Given the alpha band’s superior predictive accuracy relative to the other bands (Paired t-test: p_fdr_ < 0.01, p_fdr_ < 0.001, p_fdr_ < 0.01 for theta, beta, gamma in sertraline arm, and p_fdr_ < 0.05, p_fdr_ < 0.01 for theta, beta in placebo arm based on R^2^), all subsequent analyses in this study focused exclusively on the alpha band. Figure 2b illustrates the outcomes of the optimal predictive performance runs for the sertraline and placebo arms respectively. A substantial correlation was observed between the actual and predicted changes in HAMD_17_ scores in both arms (For sertraline: R^2^ = 0.31, Pearson’s r = 0.58, p = 5.36 × 10^−13^; For placebo, R^2^ = 0.28, Pearson’s r = 0.56, p = 1.66 × 10^−12^). The statistical significance of predictive accuracy was further validated through 1000 random permutation tests (P_permutation_<0.001 for both sertraline and placebo). Results from the other nine runs are presented in Supplementary Figure S3. To account for the influence of gender, we also regressed out gender covariate before evaluating model performance, and the results are presented in Supplementary Figure S4.

### Multimodal versus unimodal prediction

**Fig. 3:**
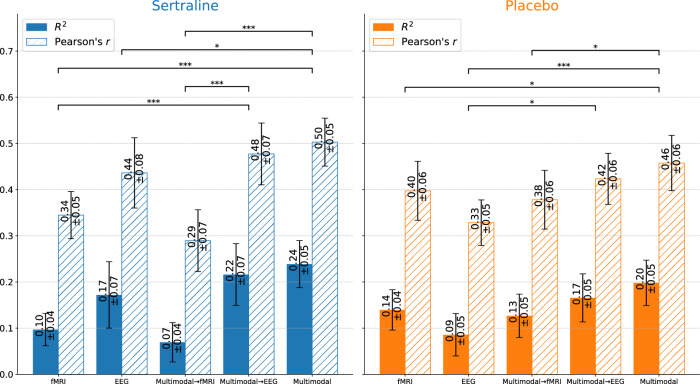
Performance comparison of treatment outcome prediction for the sertraline and placebo arms using different training and testing strategies. Blue and orange represent sertraline and placebo arms respectively. fMRI, training and testing using fMRI only. EEG, training and testing using alpha EEG only. Multimodal**→**fMRI, training with multimodal data and testing with fMRI. Multimodal${{{\boldsymbol{\to }}}}$EEG, training with multimodal data and testing with alpha EEG. Multimodal, training and testing using multimodal data.

To validate the advantage of multimodal prediction over unimodal prediction, we evaluated the performance of using fMRI and EEG independently, i.e., using unimodality for both training and testing. The model maintains its core structure, excluding only the unilateral GNN and the modality fusion component, aligning it with the original model’s design. The results shown in Fig. 3 indicate that the multimodal fusion model outperformed models trained with a single modality, whether fMRI or EEG. This finding suggests that, despite the higher cost of collecting both EEG and fMRI data in a clinical setting, it remains practically valuable for enhancing the accuracy of treatment outcome predictions and identifying more informative biomarkers.

Considering the challenges and costs of collecting both fMRI and EEG data in real-world clinical settings, we also aim to adapt our multimodal model for patients who only have unimodal neuroimaging. Our model maximizes correlation, suggesting that the latent features derived from fMRI and EEG reflect similar brain patterns while providing complementary information for treatment prediction. We trained a standalone multilayer perceptron (MLP) regressor, half the size of the original multimodal model’s MLP, using the latent features from both modalities in the training set. This allows us to use the corresponding latent features from one side of the GNN pipeline for patients with only unimodal neuroimaging, inputting them directly into the reduced MLP to generate predictions, without needing to concatenate features from the other modality. Figure 3 shows the results of training with both modalities and testing with either fMRI or alpha EEG. The data splitting and random seeds for model parameter initialization were kept identical to previous settings to ensure a fair comparison and avoid data leakage. Although predictive performance was lower with fMRI, alpha EEG yielded results only slightly inferior to multimodal prediction. This suggests that alpha EEG played a dominant role during training, with fMRI providing supplementary information. In other words, multimodal fusion provided the best predictive performance for new patients. Unimodal prediction, such as using only alpha EEG, could be improved when informed by multimodal signatures, indicating its promising clinical application value due to its much lower cost. However, it is important to note that this phenomenon may be case-specific; different training data could result in EEG not always being dominant.

### Treatment outcome-predictive brain regions

**Fig. 4:**
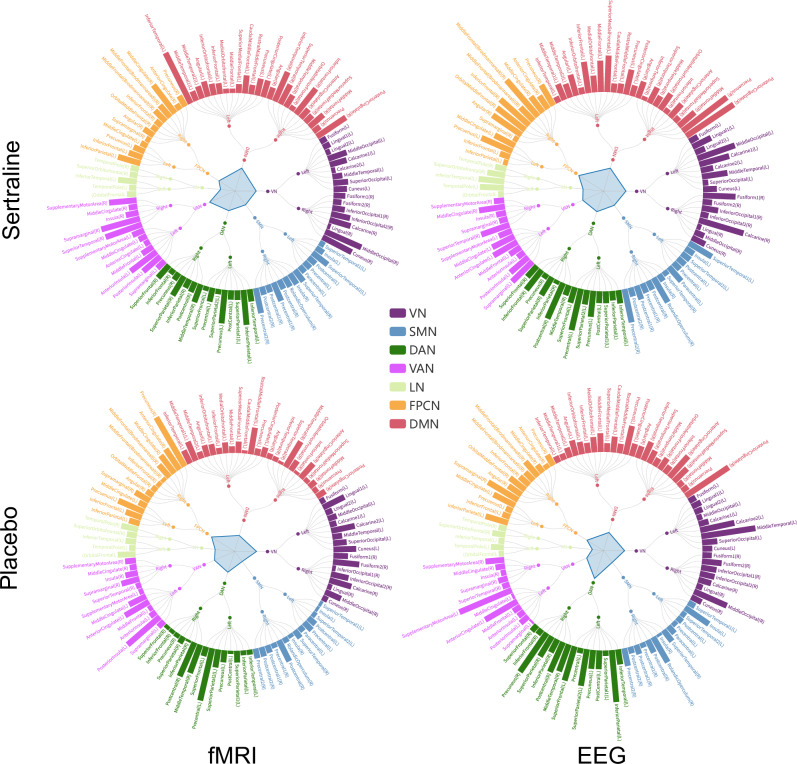
Averaged ROI’s importance scores across ten folds of the optimal run for each condition. The central radar chart reflects the importance scores of the networks by averaging the ROIs of each network. VN visual network. SMN somatomotor network. DAN dorsal attention network. VAN ventral attention network. LN limbic network. FPCN frontoparietal control network. DMN default mode network.

**Fig. 5:**
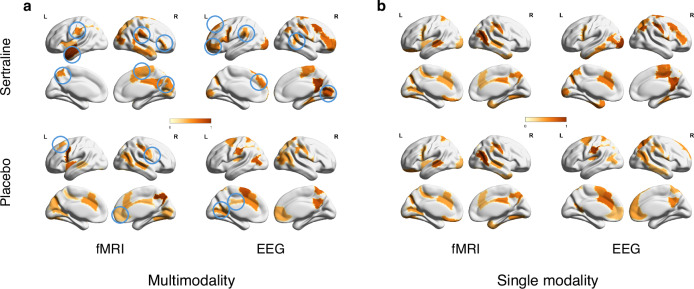
Comparison of salient ROIs between multimodal and unimodal analysis. The color reflects the importance of brain regions, with higher values indicating greater significance. **a** Top 20 salient ROIs derived from multimodal model. Blue circles identify the ROIs that are prominent in multimodal analysis but not in unimodal analysis. **b** Top 20 salient ROIs derived from unimodal model.

The ROI’s importance is presented in Fig. 4 through circular barplots. The Schaefer atlas [38] parcellated the 100 ROIs into seven spatially distributed functional networks, including visual network (VN), somatomotor network (SMN), dorsal attention network (DAN), ventral attention network (VAN), limbic network (LN), frontoparietal control network (FPCN), and default mode network (DMN). The radar chart in the center of the figure calculates the average ROI importance in each functional network, reflecting the importance at the network level. The most salient 20 ROIs with the highest weights are visualized on the cerebral cortex in Fig. 5a.

In the sertraline arm, fMRI primarily highlighted the superior and inferior temporal gyri within the temporal lobe, in addition to the middle occipital and cuneus regions in the occipital lobe, the cingulate cortex, and associative areas including the supramarginal gyrus, precuneus, and anterior insula. On the other hand, EEG pinpointed key areas in the frontal lobe, particularly in the Brodmann area 8 and inferior frontal gyrus, while also emphasizing the precuneus, posterior cingulate, calcarine, and precentral areas. In the placebo arm, fMRI predominantly revealed the precuneus, precentral, and postcentral gyrus in the parietal lobe, along with the anterior cingulate cortex, middle temporal gyrus, and posterior insula. Concurrently, EEG detected distinct regions in the frontal lobe, encompassing the supplementary motor area and anterior cingulate, as well as the precuneus, inferior parietal gyri, middle temporal, and posterior cingulate cortices. At the network level, VAN was prominently displayed in the fMRI findings, whereas FPCN was distinctly observed in the EEG data for sertraline. Moreover, FPCN was significantly represented in the fMRI, while DAN was clearly apparent in the EEG for the placebo arm.

To elucidate the advantages of multimodal approaches over unimodal ones in predicting treatment outcomes, we further explored the distinctions in salient ROIs identified by models trained separately on fMRI and EEG alone. Figure 5b displays the top 20 salient ROIs derived from a single modality. Analysis of this figure reveals that while the multimodal and unimodal models generally demonstrate similar brain patterns, notable differences exist in specific regions. In the sertraline arm, the multimodal model accentuates the inferior parietal and superior temporal regions more than the fMRI-based model, and the precentral and calcarine regions more than the EEG-based model. In the placebo arm, the multimodal model places increased emphasis on the posterior insula and rostral middle frontal regions compared to the fMRI model, and on the middle cingulate and middle occipital regions compared to the EEG model.

### Critical brain connections

**Fig. 6:**
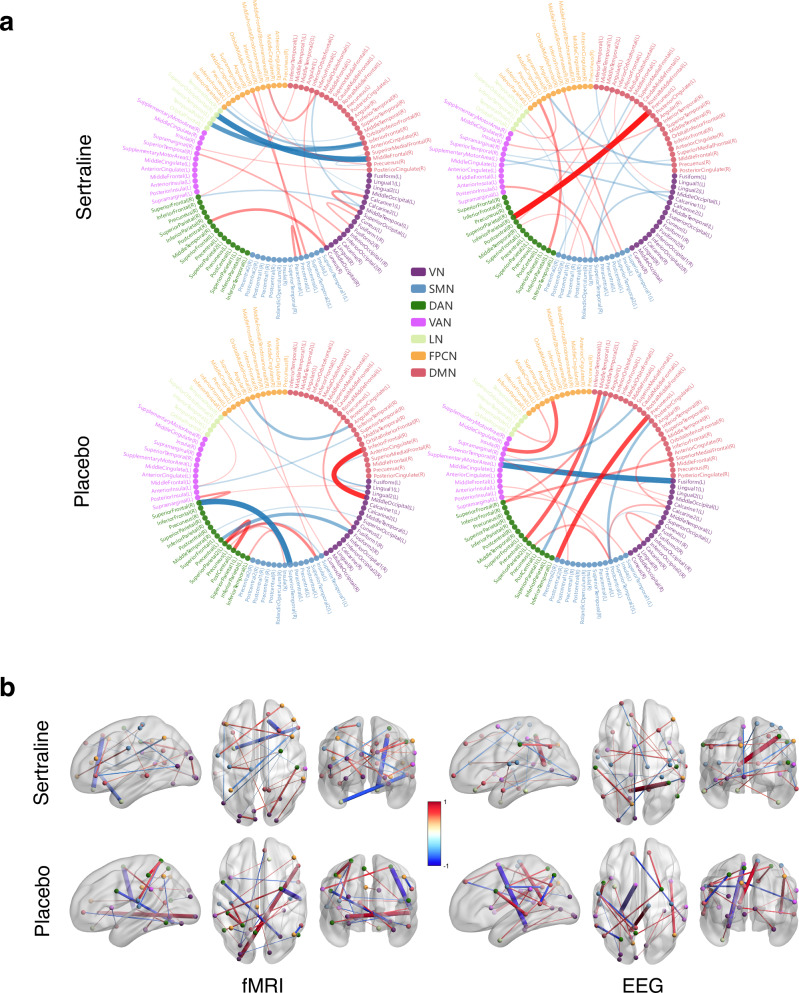
Top 20 connections with the highest averaged absolute edge scaling values. **a** Edge bunding figures for each condition. **b** Brain connectomes for each condition. Red and blue lines represent positive and negative connections respectively. The line thickness reflects the connection intensity.

The 20 most significant connections are visualized in edge bundling figures and brain connectomes in Fig. 6. In the sertraline arm, notable connections include strong negative connections between the middle frontal/superior orbitofrontal and inferior frontal/temporal pole in the fMRI connectome, and a strong positive connection between the precuneus/inferior parietal in the EEG connectome. In the placebo arm, the most prominent positive and negative connections are between the inferior frontal/middle occipital and superior temporal/superior frontal in the fMRI connectome, and between the precuneus/precentral and fusiform/supplementary motor area in the EEG connectome.

**Fig. 7:**
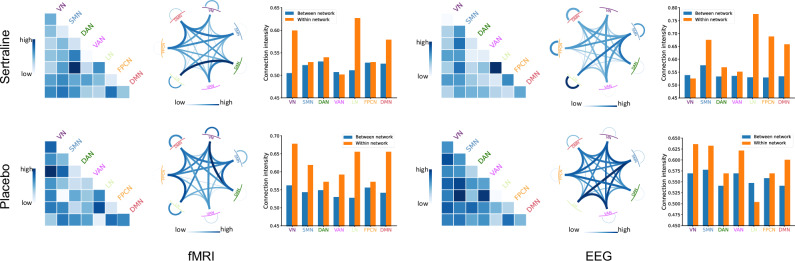
Network-level connectivity patterns. For each condition, the connection intensities of each network pair are illustrated through the heatmaps on the left and the chord charts in the middle, while the bar charts on the right represent the average between-network and within-network connection intensities of each network.

To further analyze network-level connectivity patterns, we evaluated the connection intensity by calculating the average of the absolute edge scaling values between each network pair. The results for each condition are presented using heatmaps and chord charts in Fig. 7. In the sertraline arm, LN exhibits notably high within-network connection intensity for both fMRI and EEG, with LN-DAN displaying significant inter-network connection intensity in fMRI. In the placebo arm, VN shows high within-network connection intensity for both fMRI and EEG, and VN-DAN exhibits significant inter-network connection intensity in fMRI. For EEG, LN-SMN and LN-VAN demonstrated stronger inter-network connection intensity. The bar plots in Fig. 7 represent the averaged between-network and within-network connection intensities for each condition. The between-network intensity was calculated by averaging the connection intensities across each network and the other six networks.

### Associations of brain network signatures with MDD clinical symptoms

**Fig. 8:**
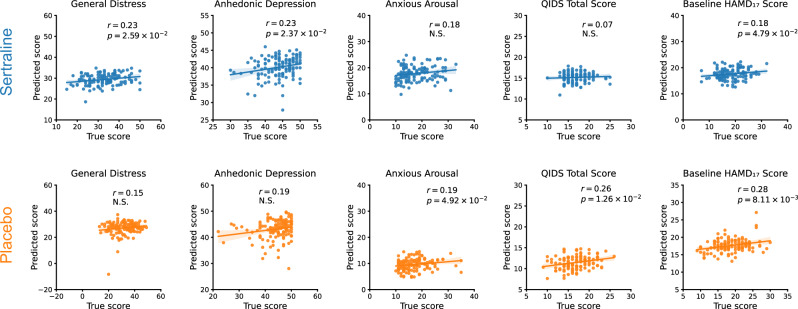
Associations of brain signatures with baseline clinical symptoms. Brain signatures of test samples were generated through previously trained multimodal models in the optimal run of 10-fold cross-validation. GAMs were then employed to predict these clinical scales using these brain signatures. *P* values are false discovery rate (FDR) corrected. N.S. means non-significant.

In the EMBARC dataset, patients with MDD were additionally evaluated using the Mood and Anxiety Symptom Questionnaire (MASQ) and the Quick Inventory of Depressive Symptomatology (QIDS). The MASQ, designed to assess mood and anxiety symptoms, categorizes conditions such as ‘General Distress’, ‘Anhedonic Depression’, and ‘Anxious Arousal’. The QIDS quantifies the severity of depressive symptoms, featuring metrics like the ‘QIDS Total Score’. We applied previously trained multimodal models to derive latent representations of test samples, which served as the inputs to the MLP regressor, subsequently using Generalized Additive Models (GAMs) [48] and conducting 10-fold cross-validation to predict these scales. As depicted in Fig. 8, the results show correlations between the clinical scales predicted by the GAMs and the actual clinical scales. In the sertraline arm, the predicted results are significantly correlated with the true scores for General Distress (r = 0.23, p = 0.026) and Anhedonic Depression (r = 0.23, p = 0.024). In the placebo arm, the predicted results are significantly correlated with the trues scores for Anxious Arousal (r = 0.19, p = 0.049) and QIDS Total Score (r = 0.26, p = 0.013). Additionally, we examined the predictability of brain signatures for baseline HAMD_17_ score. Although some correlations are observed (r = 0.18, p = 0.048 for sertraline; r = 0.28, p = 0.008 for placebo), they are notably weaker than the prediction of HAMD_17_ change (Fisher’s *z*-test p < 0.001 for sertraline and p = 0.003 for placebo). This implies that the treatment outcome prediction was not primarily driven by the baseline HAMD_17_ variation despite its correlation with brain signatures.

### Clinical implications of identified signatures

To enhance the clinical applicability of the identified predictive signatures in both treatment arms, disregarding the actual treatments received and their outcomes, we stratified all patients into either a sertraline preferred, or a placebo preferred group based on predicted responses derived from well-trained sertraline and placebo prediction models. The predictive analysis indicated 99 sertraline preferred and 31 placebo preferred patients in the sertraline arm, and 84 sertraline preferred and 51 placebo preferred patients in the placebo arm. Responder rates (defined as more than a 50% reduction in HAMD_17_ score) were significantly higher among patients whose predicted preferences aligned with their assigned treatments compared to those whose preferences did not align (Chi-square test for responder rates: sertraline arm: 57.6% [57/99] vs. 3.2% [1/31], ${\chi }^{2}$ = 26.1, *p* < 0.0001; placebo arm: 58.8% [30/51] vs. 14.3% [12/84], ${\chi }^{2}$ = 27.3, *p* < 0.0001). Similarly, actual HAMD_17_ reduction rates were significantly higher among patients whose assigned treatments matched their preferences compared to those whose treatments did not (two-sample *t*-test for treatment preference: sertraline arm: *t* = 9.5, *p* < 0.0001; placebo arm: *t* = 6.6, *p* < 0.0001). These findings provide robust clinical context for our predictions and underscore their practical relevance for treatment selection. Further details and visualizations are provided in Supplementary Figure S5.

### Ablation study

We investigated the impact of varying data augmentation rates on predictive performance, as shown in Supplementary Figure S6. Additionally, we conducted an ablation study to evaluate the effectiveness of the components within our proposed framework. The experimental results indicated that the GNNs, scaling matrices, and weight matrices were all crucial to the model. Detailed results are provided in Supplementary Table S1.

### Comparison of predictive performance with baseline methods

To demonstrate the superior predictive performance of our GNN-based framework, we also report the results of baseline methods, including SVR, Random Forest, and Elastic Net, in Supplementary Table S2. The computational complexities are summarized in Supplementary Table S3. The experimental results indicated that the baseline methods struggled to effectively predict treatment responses, even with the application of data augmentation.

## Discussion

In this study, we developed a novel GNN-based deep learning framework to integrate the functional connectivity of fMRI and EEG for identifying multimodal biomarkers of antidepressant treatment outcomes. Our model demonstrated significant efficacy in predicting responses to both sertraline and placebo. The GNNs effectively mapped interactions between ROIs, yielding spatially optimized representations for fMRI and EEG connectivity. By leveraging adaptive edge scaling matrices that dynamically refine connectivity strength, the model improved modality fusion and prediction accuracy. Integrating correlations between GNN-encoded latent features allowed the extraction of informative multimodal brain patterns, revealing synergistic information from both fMRI and EEG. Importantly, we adapted our model for situations where patients have only EEG data by training a standalone MLP, which yielded more accurate predictions than models trained with a single modality. This adaptation greatly enhances the model’s practical utility in real clinical settings. The findings indicate that multimodal fusion provided the best predictive performance, underscoring the value of combining EEG and fMRI data despite the higher costs. The adaptation for unimodal prediction, using only alpha EEG, showed promise by leveraging multimodal signatures. This approach maintains clinical application value due to its lower cost, although it is important to recognize that the dominance of EEG in predictive performance may be case-specific. Different training data or prediction tasks could result in scenarios where EEG is not always the dominant modality. Further research should explore these dynamics to optimize the use of unimodal in clinical settings. On the other hand, predicting placebo treatment, while not directly relevant to clinical care, holds considerable potential in drug development. A placebo-predictive signature could enhance phase 2 studies by selecting comparator groups with low predicted placebo responses rates, potentially reducing costs by allowing for smaller sample sizes and more robust drug efficacy assessments.

Our model not only delivers impressive predictive performance but also offers valuable insights through interpretative analysis. By examining the learnable parameters, including edge scaling matrices for spatial pattern optimization and weight matrices for modality fusion, we identified common brain patterns across modalities and biomarkers specific to antidepressant sertraline or placebo. This analysis revealed distinct ROIs and connectivity patterns associated with each treatment, enhancing our understanding of the neural circuits involved. Our findings align with previous research, indicating that connectivity across theta, alpha, and gamma bands in EEG provides essential insights into treatment responses [4, 49–51]. We observed that the prefrontal cortex, cingulate cortex, and specific areas of the temporal and parietal lobes provide signals crucial for predicting antidepressant response, which are consistent with findings reported in previous studies [52–55]. Notably, the superior temporal gyrus and posterior cingulate cortex substantially contributed to sertraline response in both fMRI and EEG modalities (Fig. 4). These regions are linked to emotional regulation and social cognition, critical factors in depression [56–62]. For predicting placebo response, the anterior cingulate cortex and the postcentral gyrus played consistent roles across modalities. Elevated resting activity in this region may indicate better clinical outcomes, as it is linked to adaptive self-referential processing and enhanced cognitive control [63]. Furthermore, numerous studies have demonstrated the association between the anterior cingulate cortex-involved functional connectivity changes and placebo response [49, 64–66]. Additionally, the postcentral gyrus, associated with emotional regulation and sensory processing, has also been implicated in various forms of depression [67, 68]. Moreover, our multimodal approach uniquely identifies specific brain regions compared to the unimodal approach, such as the superior temporal gyrus on fMRI and the posterior insula on EEG. These findings underscore the advantages of multimodal fusion, which uncovers associations that are not detectable through single-modality analysis. The posterior insula, in particular, is crucial for emotion processing [69] and has been extensively implicated in the pathophysiology of depression [70–73].

At the network level, our findings (Fig. 4) indicate that FPCN, VAN, and DAN are essential for predicting treatment responses from a multimodal integration perspective. FPCN, involved in problem-solving, working memory, and emotion regulation [74, 75], was prominent in the EEG modality for the sertraline arm and the fMRI modality for placebo. VAN, responsible for attention switching and integrating emotional and sensory stimuli [76–78], along with DAN, involved in goal-directed external attention [79], suggests that the extent to which individuals with MDD respond to antidepressants or placebo may depend on externally oriented attention and processing salience. Figure 7 shows that FPCN typically exhibits weaker within network connections while DMN, associated with internally oriented attention and self-referential thought, shower stronger within-network connections compared to those of DAN. Strong inter-network connections between FPCN and DMN may underlie cognitive control deficits observed in MDD [70], such as difficulties in concentration and emotion regulation. Although LN comprises fewer brain regions, it features multiple high-intensity interconnections and exhibits the strongest within-network connections in the sertraline arm. The LN system has consistently been associated with the control of emotions and mood disorders, autonomic regulation, and cognitive deficits [80], and shown sensitivity to stress correlates with the severity of depression [81–83]. These specific network patterns may contribute to core deficits in cognitive and affective functioning, thereby serving as brain signatures to inform treatment outcomes. Our framework provides additional validation for the importance of specific brain regions, functional networks, and connections as biomarkers in predicting treatment outcomes for MDD, offering a new perspective on neurobiological mechanisms and underscoring their potential utility in real-world diagnostic scenarios.

Our results also highlight significant correlations between signature-predicted baseline clinical scores from GAMs and the actual ones in both the sertraline and placebo arms, providing supplementary evidence to confirm the clinical relevance of the identified brain network signatures. The use of the MASQ and QIDS scales in this study underscores their importance in capturing the complex symptomatology of MDD. The correlations found with these scales provide a nuanced understanding of how symptoms, such as anhedonia and anxiety, are reflected in brain network function. This insight could enhance diagnostic criteria and aid in the creation of targeted interventions for these symptoms. Future studies might investigate the stability of these correlations through longitudinal research and across different treatment arms. Moreover, extending the analysis to other forms of depression and anxiety disorders could reveal broader applications of these brain network signatures.

This study has several limitations and potential areas for expansion that merit consideration. Despite our study having a larger sample size compared to most existing clinical trial studies on antidepressant treatment, the data remains modest for training deep learning models. This limitation was highlighted by our data augmentation ablation analysis. Thus, to validate our biomarker findings, larger sample sizes and replication studies with independent cohorts are essential [84]. Currently, our prediction model is based on baseline data; future research should consider longitudinal data to better understand the relationship between functional connectivity changes and treatment outcomes. Expanding our modality fusion approach to integrate more data types will also provide richer neurophysiological information. Additionally, with larger sample sizes, advanced deep learning techniques could be explored to handle partial modality missing, which is common in clinical settings. Promising approaches include variational autoencoders for learning shared latent representations across modalities [85], self-attention mechanisms for flexible modality fusion [86], and contrastive learning strategies to align representations from different modalities even with incomplete data [87]. These approaches hold promise for improving model robustness and generalizability when faced with real-world data heterogeneity.

## Conclusion

This study aimed to predict treatment outcomes in MDD and quantify brain signatures using a GNN-based deep learning method that integrates fMRI and EEG data. Our approach identified key brain regions and connections related to antidepressant response, highlighting the utility of multimodal approach in understanding complex brain interactions. Leveraging data from the EMBARC clinical trial, along with advanced data augmentation and modality fusion techniques, the framework enhanced prediction performance and revealed neurobiological underpinnings of MDD. Our interpretability analysis underscored crucial regions: the inferior temporal gyrus and posterior cingulate for sertraline, and the precuneus and supplementary motor area for placebo responses. The superior temporal gyrus and posterior cingulate cortex were consistently significant across both modalities in the sertraline arm, while the anterior cingulate cortex and postcentral gyrus were notably significant in the placebo arm, underscoring their fundamental role in predicting antidepressant and placebo efficacy. Our findings emphasize the importance of large-scale brain networks, such as the frontoparietal control, ventral and dorsal attention, and limbic networks, in MDD treatment dynamics. Overall, our research enriches the understanding of antidepressant medications in psychiatry through multimodal neuroimaging, offering novel insights on mental health treatment.

## Supplementary information

Supplementary materials

## Data Availability

The COBE augmentation was implemented in MATLAB (v.R2024a). The deep learning framework was implemented in Python (v.3.11.10) and PyTorch (v.2.1.1). The statistical analyses were conducted using the SciPy package (v.1.11.4). The code used in this study is available at https://github.com/YongJiao10/MultimodalGraph4MDD.
