# Supplementary material for: Deep graph learning of multimodal brain networks defines treatment-predictive signatures in major depression
Source: Mol Psychiatry. 2025 Mar 31;30(9):3963–74. doi: 10.1038/s41380-025-02974-6 (PMC12339373; doi:10.1038/s41380-025-02974-6)
Supplement: Supplementary file 1 — Supplementary materials [file 41380_2025_2974_MOESM1_ESM.docx]

**Supplementary Material**

**Supplementary Methods**

**[S1. Common orthogonal basis extraction (COBE)](#S1)**

[Figure S1. Illustration of the COBE based data augmentation](#FigS1)

### [**S2. Deep Learning of Multimodal Brain Network Signatures**](#S2)

[Figure S2. The flowchart of the framework.](#FigS2)

[**S3. Experimental Setup**](#S3)

**Supplementary Results**

**[S4. Treatment Outcome Prediction](#S4)**

[Figure S3. The other four runs of prediction results.](#FigS3)

[Figure S4. Performance of treatment outcome prediction with gender regressed out.](#FigS4)

**[S5. Experiments](#S5)**

[Figure S5. The efficacy of identified predictive signatures in treatment stratification.](#FigS5)

[Figure S6. The impact of augmentation rate on prediction performance.](#FigS6)

[Table S1. Ablation study of learning strategies on our proposed deep learning framework.](#table1)

[Table S2. Comparison of predictive performance with baseline methods.](#table2)

[Table S3. Computational complexity analysis of different methods.](#table3)

**Supplementary Methods**

### **S1. Common orthogonal basis extraction (COBE)**

COBE is a well-established algorithm in group component analysis to extract the common basis from multi-block data (1). Given the multi-block data $\mathcal{X=}\left\{ \mathbf{X}_{k}\in\mathbb{R}^{{D\times N}_{k}}:k=1, 2,...,K \right\}$, where $N_{k}$ represents the number of samples in the $k$-th group and $D$ the number of features. COBE decomposes the data into common components and individualized features by solving the optimization problem:

$$\min_{\bar{\mathbf{A}}, {\breve{\mathbf{A}}}_{k}} \sum_{k=1}^{K} \left. ||\mathbf{X}_{k}-\bar{\mathbf{A}}{\bar{\mathbf{B}}}_{k}^{T}-{\breve{\mathbf{A}}}_{k}{\breve{\mathbf{B}}}_{k}^{T} \right.||_{F}^{2}$$

$$s.t. {\bar{\mathbf{A}}}^{T}\bar{\mathbf{A}}=\mathbf{I},{\breve{\mathbf{A}}}_{k}^{T}{\breve{\mathbf{A}}}_{k}=\mathbf{I}, {\bar{\mathbf{A}}}^{T}{\breve{\mathbf{A}}}_{k}=\mathbf{0}$$

where $C$ columns of $\bar{\mathbf{A}}\in\mathbb{R}^{D\times C}$ represent the shared latent variables, and ${\bar{\mathbf{B}}}_{k}\in\mathbb{R}^{N_{k}\times C}$ denotes the corresponding coefficients. By removing the common components $\bar{\mathbf{A}}{\bar{\mathbf{B}}}_{k}^{T}$ from the original data $\mathbf{X}_{k}$, individualized features ${\breve{\mathbf{A}}}_{k}{\breve{\mathbf{B}}}_{k}^{T}$ are isolated. These individualized features, as newly generated data, retain critical discriminatory information pertinent to their original samples, thereby forming the augmented data in this study. Specifically, the augmented training samples ${\hat{\mathbf{X}}}_{k}$ are obtained by:

$${\hat{\mathbf{X}}}_{k}=\mathbf{X}_{k}-\bar{\mathbf{A}}{\bar{\mathbf{B}}}_{k}^{T}$$

while the testing samples $\mathbf{Y}$ can be augmented via:

$$\hat{\mathbf{Y}}=\mathbf{Y}-\bar{\mathbf{A}}{\bar{\mathbf{A}}}^{T}\mathbf{Y}$$

When the number of decomposed components $C$ is set to $1, 2,...,T$, $T$ sets of augmented data are generated.

**Figure S1. Illustration of the COBE based data augmentation.** The FCs of all subjects were initially randomly divided into K groups. COBE then extracted $C (C=1, 2,...,T)$ common components for each group. Individualized FCs were obtained by subtracting common components from raw FCs, forming the augmented data.


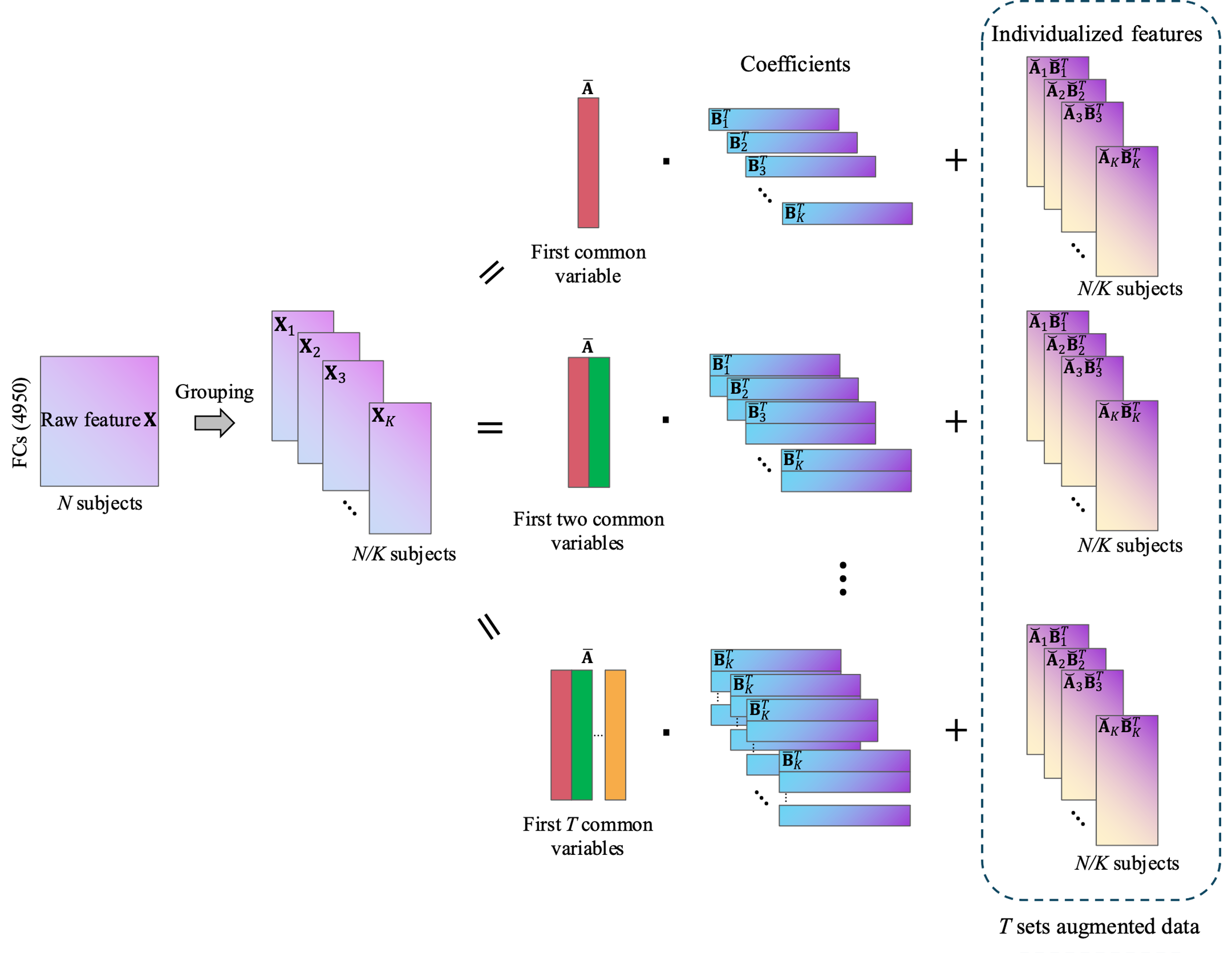


**S2.** **Deep Learning of Multimodal Brain Network Signatures**

Given the FCs of fMRI and EEG $\mathbf{X}_{\mathrm{fMRI}}, \mathbf{X}_{\mathrm{EEG}}\in\mathbb{R}^{R\times R}$, where $R$ denotes the number of ROIs. The undirected graphs could be constructed by treating rows of FCs $\left\{ \mathbf{x}_{i}\in\mathbb{R}^{1\times R}:i=1, 2,...,R \right\}$ as the node features, and dynamically calculating the weighted adjacent matrix $\mathbf{E}^{\mathcal{l}}=[e_{ij}^{\mathcal{l}}]\in\mathbb{R}^{R\times R}$ in the $\mathcal{l}$-th GNN layer by:

$$e_{ij}^{\mathcal{l}}=cos(\mathbf{h}_{i}^{\mathcal{l}},\mathbf{h}_{j}^{\mathcal{l}})$$

where $\mathbf{h}_{i}^{\mathcal{l}}\in\mathbb{R}^{1\times d}$ denotes the $i$-th node feature in the $\mathcal{l}$-th layer ($\mathbf{h}_{i}^{0}=\mathbf{x}_{i}$), $cos(\cdot)$ represents cosine similarity. This way, edge can accurately and in real-time reflect the connection strength between nodes.

Considering the heterogeneity inherent in multi-modal neuroimaging, direct aggregation of features could potentially deteriorate performance. A crucial aspect lies in precisely interpreting the brain network pattern specific to each modality. Thus, we utilized parallel GNNs to encode the graph pairs formed by fMRI and EEG FCs. The message passing in the $l$-th layer in each GNN can be described as follows:

$$\mathbf{H}^{\mathcal{l+}1}=(\mathbf{S}⨀\mathbf{E}^{\mathcal{l}})\mathbf{H}^{\mathcal{l}}\mathbf{W}^{\mathcal{l}}$$

where $\mathbf{W}^{\mathcal{l}}$ represents a trainable weight matrix enhancing node representations, $\mathbf{H}^{\mathcal{l}}$ signifies node features, and $\mathbf{S}$ is a symmetric learnable scaling matrix. This matrix is designed to emphasize the significance of message transmission between node pairs. By undergoing the element-wise multiplication with $\mathbf{E}^{\mathcal{l}}$, the system selectively strengthens node connections, guided by the demands of the prediction task. To reduce irrelevant connections to negligible levels, we implement sparsity on $\mathbf{S}$ by imposing $L_{1}$ norm constraint within the edge loss formulation:

$$\mathcal{L}_{edge}=\left\| \mathbf{S}_{\mathrm{fMRI}} \right\|_{1}+\left\| \mathbf{S}_{\mathrm{EEG}} \right\|_{1}$$

To align the GNN encoded features $\mathbf{H}_{\mathrm{fMRI}}$ and $\mathbf{H}_{\mathrm{EEG}}\in\mathbb{R}^{R\times d}$ for effective fusion, we introduce two sets of learnable weight vectors $\mathbf{U}=\left[ \mathbf{u}_{1},\mathbf{u}_{2},...,\mathbf{u}_{P} \right]$, $\mathbf{V}=\left[ \mathbf{v}_{1},\mathbf{v}_{2},...,\mathbf{v}_{P} \right]\in\mathbb{R}^{R\times P}$ to linearly combine the respective node dimensions, with each latent variable pair representing a multimodal brain pattern. We promote the enhancement of correlations between these patterns by incorporating a correlation loss defined as:

$$\mathcal{L}_{corr}=-\sum_{p=1}^{P} corr(\mathbf{H}_{\mathrm{fMRI}}^{T}\mathbf{u}_{p},\mathbf{H}_{\mathrm{EEG}}^{T}\mathbf{v}_{p})$$

where $corr(\cdot)$ computes Pearson’s correlation. Through backpropagation, $\mathbf{U}$ and $\mathbf{V}$ are iteratively refined, selectively emphasizing nodes conducive to modality fusion and the target predictive task. To further improve the model's interpretability, we also introduce sparsity on $\mathbf{U}$ and $\mathbf{V}$, resulting in a node loss expressed as:

$$\mathcal{L}_{node}=\left\| \mathbf{U} \right\|_{1}+\left\| \mathbf{V} \right\|_{1}$$

The final representation of the multimodal graph pair can be summarized by:

$$\mathbf{h}_{\mathcal{G}}=\sum(\mathbf{U}^{T}\mathbf{H}_{\mathrm{fMRI}}\parallel\mathbf{V}^{T}\mathbf{H}_{\mathrm{EEG}})⨀\mathbf{z}$$

Here, $\parallel$ is concatenation operator, and $\mathbf{z}\in\mathbb{R}^{P}$ represents weighted values of $P$ brain patterns, learned during model training. Following this, $\mathbf{h}_{\mathcal{G}}$ serves as input for a multilayer perceptron (MLP) regressor, tasked with predicting the change in HAMD_17_ score. The final loss function of the model is formulated as:

$$\mathcal{L} = \mathcal{L}_{MSE}+\lambda_{1}\mathcal{L}_{corr}+\lambda_{2}\mathcal{L}_{edge}+\lambda_{3}\mathcal{L}_{node}$$

In this equation, $\mathcal{L}_{MSE}$ is mean square error loss and $\lambda_{i} (i=1,2,3)$ are adjustable hyperparameters that act as penalty coefficients for various loss terms. Our approach, through the use of sparse scaling matrices and weight vectors, highlights key connections and prominent ROIs in both modalities, essential for predicting changes in HAMD_17_ score.

Figure S2. The flowchart of the framework. FCs from fMRI and EEG are firstly obtained based on parcellation and encoded using two parallel GNNs, with a learnable edge scaling matrix set in the GNNs to optimize spatial patterns. Then, the encoded multimodal representations are projected onto latent variables using two learnable weight matrices. The correlation coefficients between the latent variables are incorporated into the loss function to enhance their compatibility. The latent variables are concatenated and feature-aggregated using a one-dimensional convolution before being fed into an MLP to predict changes in HAMD_17_ score and calculate MSE loss. Finally, model parameters are optimized through backpropagation.


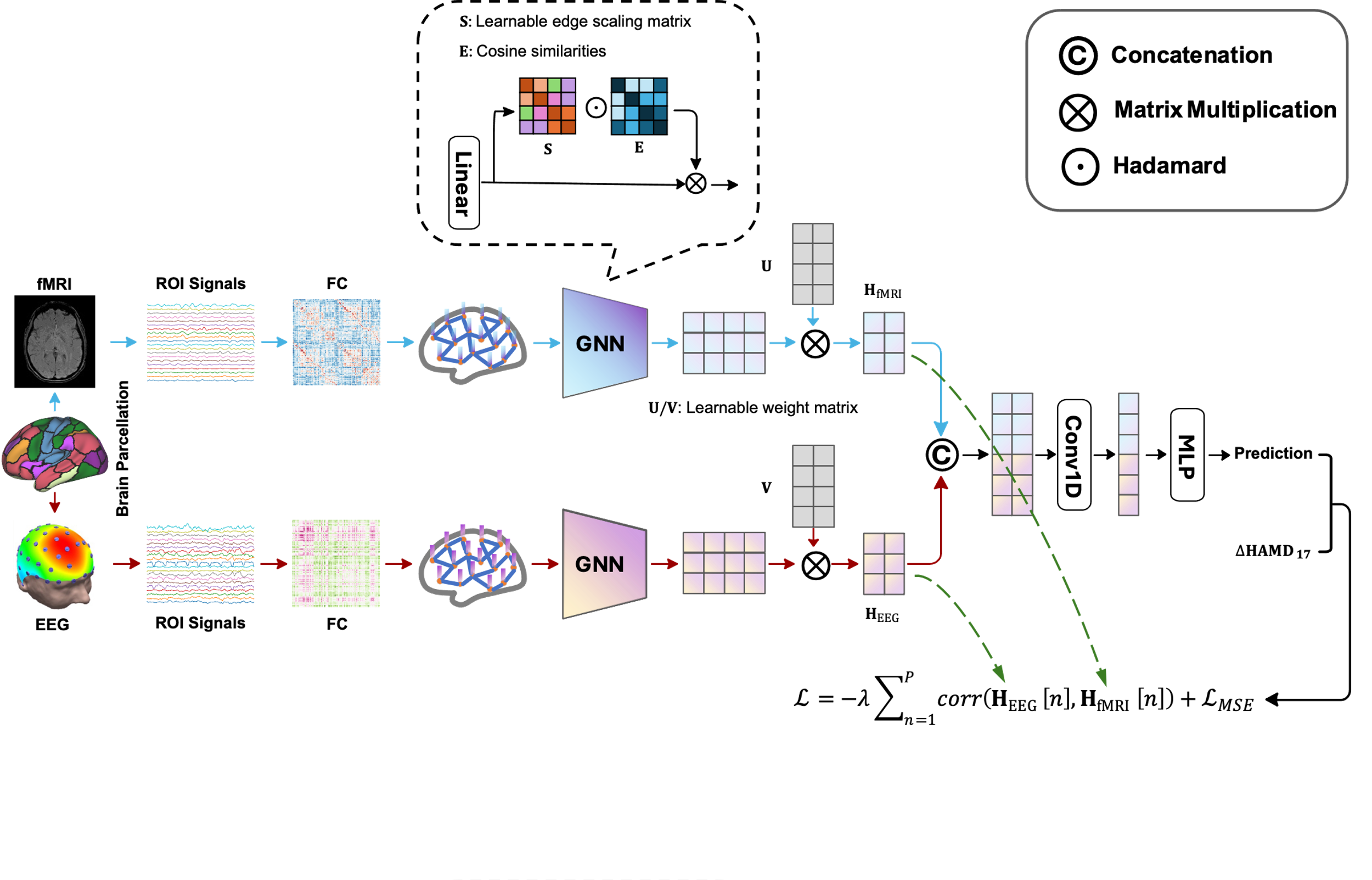


**S3. Experimental Setup**

In data augmentation, patients receiving two treatments were collectively analyzed to derive common components through COBE decomposition, given the unsupervised nature of the algorithm. We employed 10-fold cross-validation to evaluate prediction performance. Specifically, in the experiment for a certain treatment, data augmentation was conducted independently for each fold to prevent information leakage, extracting common components from the collection that included both training samples and all samples from the alternate treatment. The number of groups $K$ was set to 10 following (2,3). Considering the Schaeffer atlas, with $R=100$ ROIs, the number of features $D=100\times(100-1)/2=4950$. The iteration number $T$ was fixed at 4, quintupling the total sample size due to the creation of four augmented samples per real sample.

Our study utilized a parallel dual-layer graph convolutional network with a hidden layer size $d=50$. The matrices $\mathbf{U}$ and $\mathbf{V}$ contained $P=16$ learnable weight vectors. The MLP dimensions were set to [128, 32, 1], incorporating a 0.5 dropout rate to mitigate overfitting. We used an Adam optimizer with a learning rate of 0.006, a batch size of 64, and penalty coefficients $\lambda_{1}$, $\lambda_{2}$, and $\lambda_{3}$ of 2, 0.001, and 0.05, respectively. The framework was implemented in Pytorch on an NVIDIA GeForce RTX 4090.

**S4. Treatment Outcome Prediction**

**Figure S3. The other nine runs of predictive outcomes for the sertraline and placebo arms respectively.** Each point represents a patient in the test set.

Sertraline

Placebo


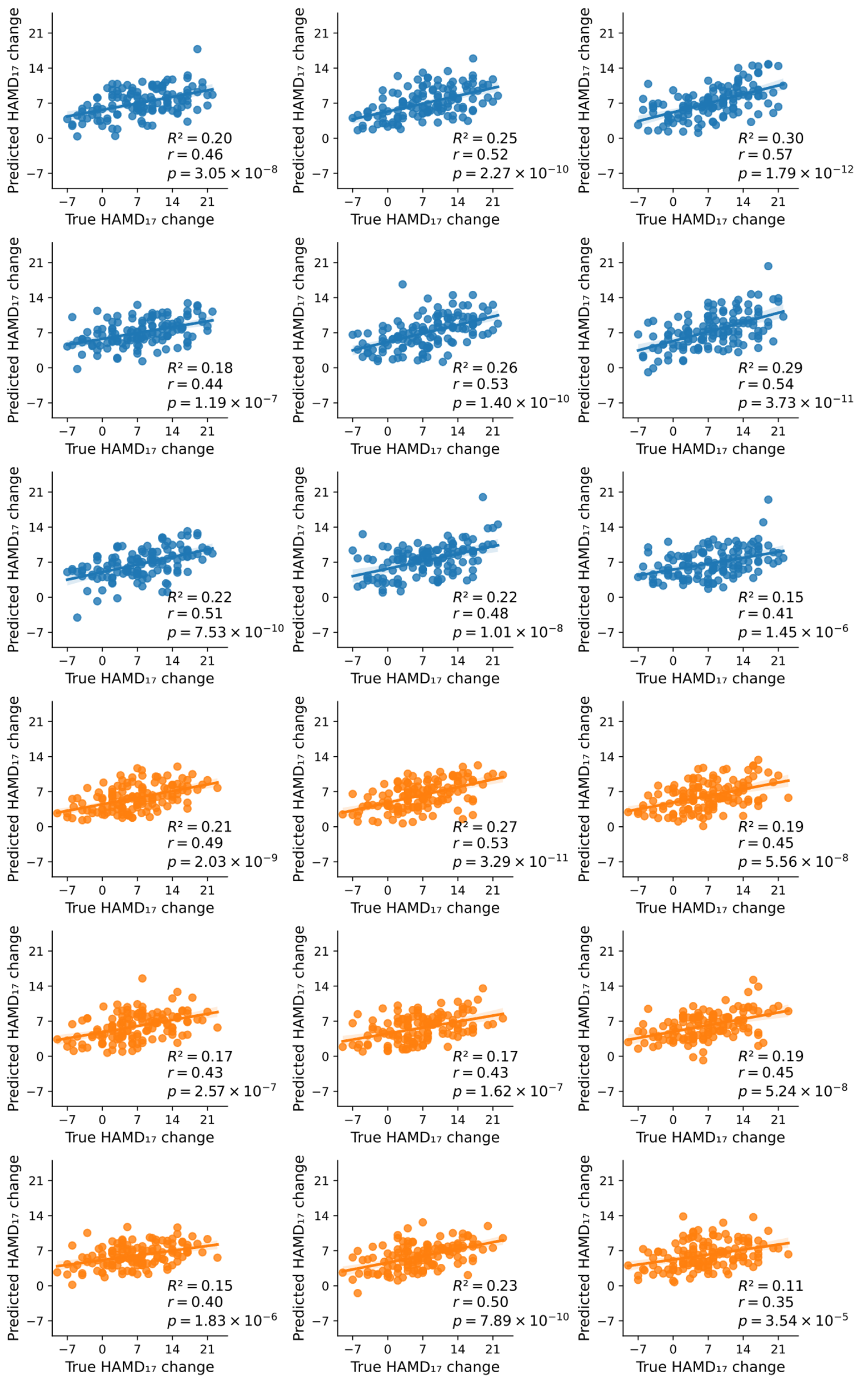


**Figure S4. Performance of treatment outcome prediction with gender regressed out. a.** Prediction results across four EEG bands under our deep learning-based multimodal analytical framework. **b.** The optimal run of predictive outcomes using alpha band EEG in conjunction with fMRI. Each point represents a patient in the test set.


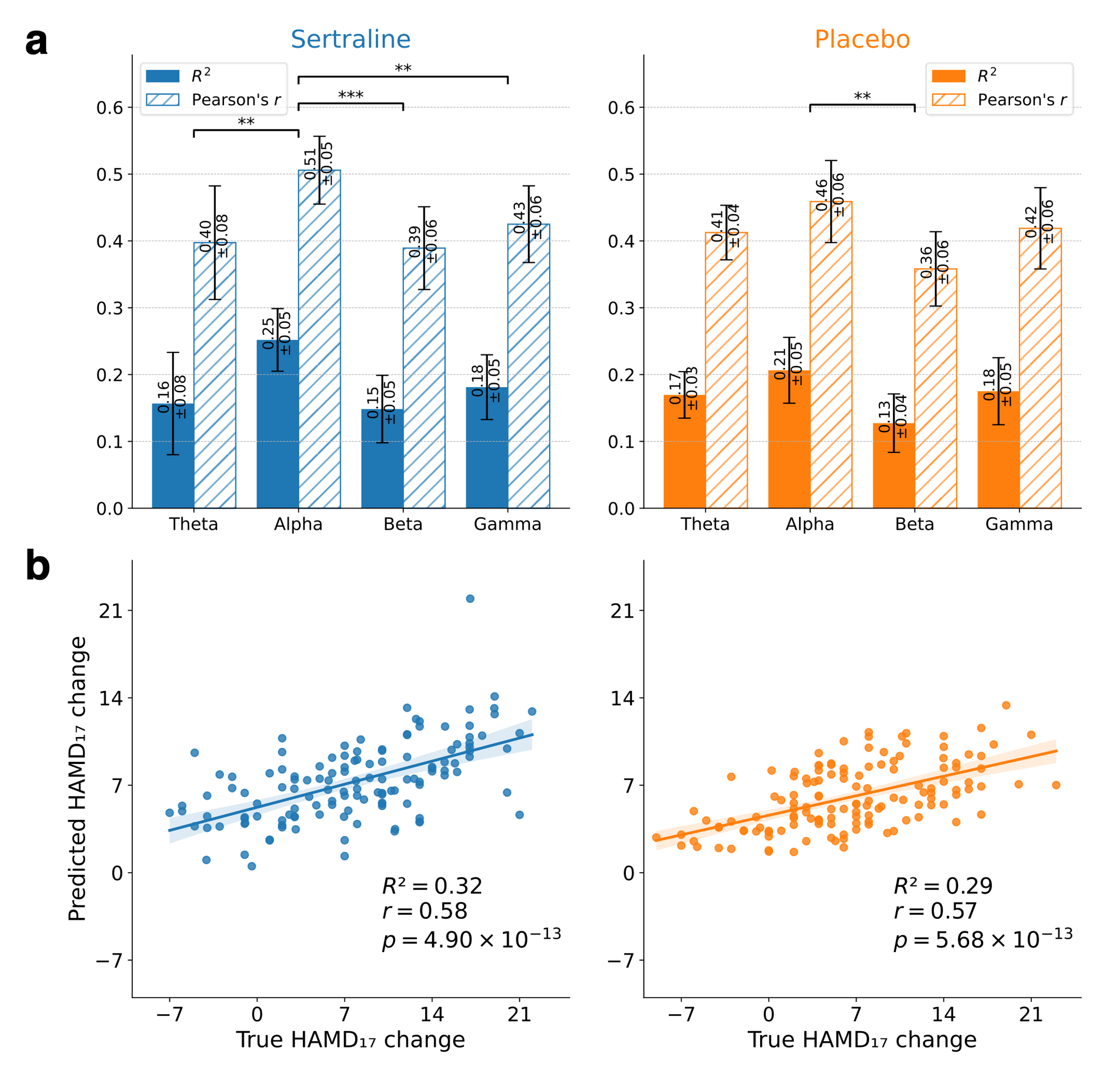


**S5. Experiments**

**Figure S5. The efficacy of identified predictive signatures in treatment stratification. a.** Predicted sertraline and placebo response obtained by treatment-specific prediction models for each patient. Patients with a higher predicted response to sertraline were categorized as "sertraline preferred," while those with a higher predicted response to placebo were designated as "placebo preferred." **b**. Responder rates by treatment preference in each treatment arm. A responder was defined as a patient achieving an actual reduction of more than 50% in HAMD_17_ score. Responder rates among patients assigned to their preferred treatment were significantly higher than those assigned to non-preferred treatment (Chi-square test for treatment preferences: sertraline arm: $\chi^{2}=26.1, p<0.0001$; placebo arm: $\chi^{2}=27.3, p<0.0001$). **c**. Actual HAMD_17_ reduction rates by treatment preference in each treatment arm. Patients assigned to their preferred treatment exhibited significantly higher reduction rates than those assigned to non-preferred treatment (Two-sample *t*-test for treatment preference: sertraline arm: $t=9.5, p<0.0001$; placebo arm: $t=6.6, p<0.0001$).


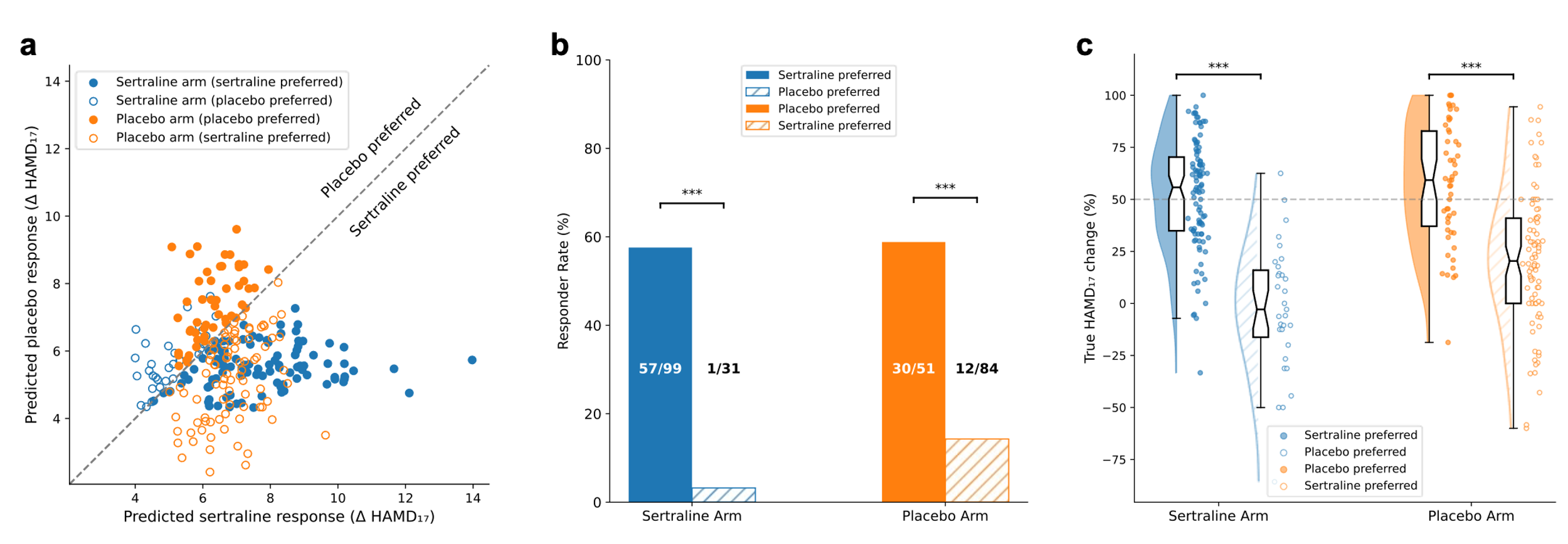


**Figure S6. The impact of augmentation rate on prediction performance.** A rate of 0 indicates the exclusive use of original data without augmentation. It is observed that the performance significantly improves as the rate increases, but there is no further improvement beyond a rate of four for sertraline and two for placebo. Therefore, we chose the augmentation rate $T=4$.


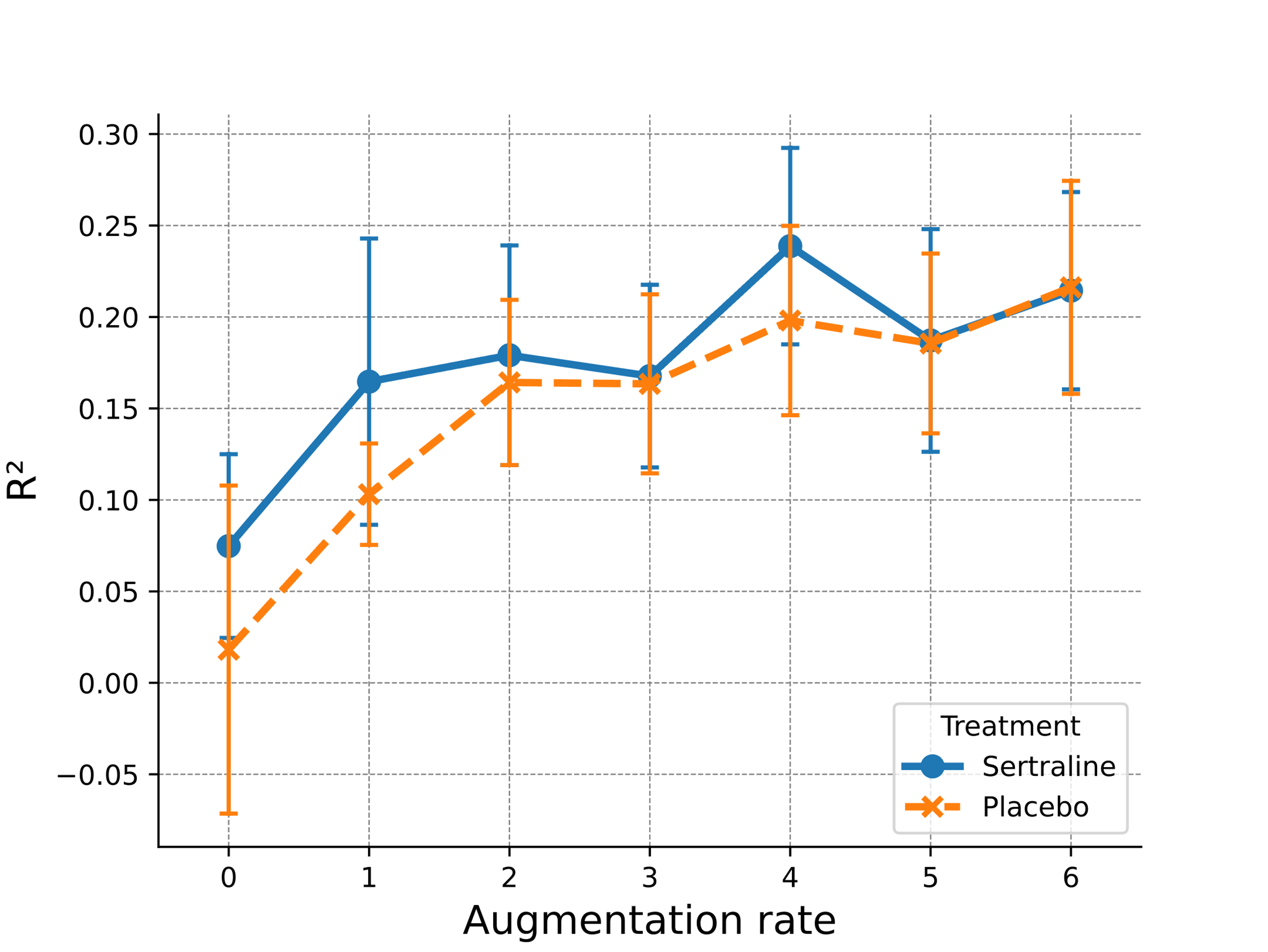


**Table S1. Ablation study of learning strategies on our proposed deep learning framework.** The results illustrate the impact of removing elements such as the GNNs, scaling matrices, and correlation loss. The removal of the GNNs led to a notable reduction in performance, underscoring its pivotal role in accurately interpreting the complex interconnections within brain networks. The scaling matrix proved to be crucial either, suggesting that the construction of a graph's adjacency matrix should not solely rely on the data's intrinsic attributes. Instead, task-specific adaptive spatial optimization is essential. In addition, merging modalities without maximizing correlation is unable to fully leverage the complementary information between them.

|  | Sertraline | | Placebo | |
| --- | --- | --- | --- | --- |
|  | $R^{2}$ | Pearson’s r | $R^{2}$ | Pearson’s r |
| w/o GNNs | 0.14±0.06 | 0.39±0.06 | 0.03±0.09 | 0.26±0.11 |
| w/o Scaling Matrices | 0.03±0.10 | 0.23±0.14 | 0.10±0.04 | 0.39±0.04 |
| w/o Correlation | 0.18±0.02 | 0.44±0.02 | 0.16±0.06 | 0.41±0.06 |
| Full model | **0.24±0.05** | **0.50±0.05** | **0.20±0.05** | **0.46±0.06** |

**Table S2. Comparison of predictive performance with baseline methods.** For the baseline methods, we first applied PCA to both fMRI and EEG data separately, selecting 40 components for each after conducting a grid search. The reduced feature sets were then concatenated, resulting in samples with 80 features, which were subsequently fed into the baseline models. The COBE data augmentation strategy was applied uniformly. The results indicate that only SVR and Elastic Net demonstrated modest predictive performance in the sertraline arm.

|  | Sertraline | | Placebo | |
| --- | --- | --- | --- | --- |
|  | $R^{2}$ | Pearson’s r | $R^{2}$ | Pearson’s r |
| SVR | 0.05 | 0.24 | -0.05 | 0.07 |
| Elastic Net | 0.05 | 0.31 | -0.32 | -0.04 |
| Radom Forest | 0.00±0.00 | 0.18±0.01 | -0.13±0.00 | -0.10±0.01 |
| Ours | **0.24±0.05** | **0.50±0.05** | **0.20±0.05** | **0.46±0.06** |

**Table S3. Computational complexity analysis of different methods.** Complexity analysis for both training and prediction phases for various methods. The dataset's sample size is denoted by *n*, while the feature dimensions are represented as *D* (4950 in our case, same for the follows) for the original input features of each modality and *k* (40) for the reduced dimensions obtained after PCA transformation. Specific model parameters include $n_{sv}$​ (approximately 1000), representing the number of support vectors in SVR; $n_{trees}$ ​(100), indicating the number of trees constructed in the Random Forest algorithm; and *T* (approximately 30), denoting the number of iterations required for Elastic Net convergence. In our GNN architecture, *R* (100) refers to the number of nodes, *L* (2) indicates the number of graph neural network layers, *d* (50) specifies the hidden dimension size, *P*​ (16) is the number of learnable weight vectors, *E* represents the number of training epochs, and *b* (64) denotes the batch size used during training.

|  | Training Complexity | Prediction Complexity |
| --- | --- | --- |
| PCA + SVR | $O(nD^{2})+O(D^{3})+O(n^{3})$ | $O\left( Dk \right)+O(n_{sv}k)$ |
| PCA + Elastic Net | $O(nD^{2})+O(D^{3})+O(nkT)$ | $O\left( Dk \right)+O(k)$ |
| PCA + Random Forest | $O(nD^{2})+O(D^{3})+O(n_{trees}nlog(n))$ | $O\left( Dk \right)+O(n_{trees}log(n))$ |
| Ours | $O(E\lceil n/b\rceil(LR^{2}d+RPd))$ | $O(LR^{2}d)+O(Rd)+O(d^{2})$ |

**References**

1. Zhou G, Cichocki A, Zhang Y, Mandic DP. Group Component Analysis for Multiblock Data: Common and Individual Feature Extraction. IEEE Transactions on Neural Networks and Learning Systems. 2016 Nov;27(11):2426–39.

2. Sagonas C, Ververas E, Panagakis Y, Zafeiriou S. Recovering Joint and Individual Components in Facial Data. IEEE Trans Pattern Anal Mach Intell. 2018 Nov 1;40(11):2668–81.

3. Zhao K, Xie H, Fonzo GA, Tong X, Carlisle N, Chidharom M, et al. Individualized fMRI connectivity defines signatures of antidepressant and placebo responses in major depression. Mol Psychiatry. 2023; 28(6): 2490–2499.
